# CHILDHOOD SOCIOECONOMIC STATUS AND SENSE OF CONTROL OVER COGNITIVE AGING: DO GENES MODERATE?

**DOI:** 10.1093/geroni/igz038.3227

**Published:** 2019-11-08

**Authors:** Addam S Reynolds, Emily Greenfield

**Affiliations:** 1 Rutgers University School of Social Work, New Jersey, United States; 2 Rutgers University, New Brunswick, New Jersey, United States

## Abstract

Individuals who lack a sense of control over cognitive aging (SOC-CA) believe little can be done to optimize their cognitive functioning. While prior research indicates that higher SOC-CA is a protective factor against age-related cognitive decline, few studies have examined predictors of change in SOC-CA. To address this gap, we used data from the Midlife in the United States (MIDUS) study. Guided by prior research on linkages between socioeconomic status (SES) and control beliefs, we examined childhood SES as an early life course influence on changes in SOC-CA. The analytic sample consisted of 663 White participants, ages 34 to 81, who were interviewed in 2004 and approximately nine years later. SOC-CA was measured by using three items from the Personality in Aging Context scale, and childhood SES encompassed retrospective reports of parental education and occupational status. A hierarchical linear model was estimated, which modeled SOC-CA at baseline, as well as change over the study period, controlling for gender, age, ancestry, and adult SES. While childhood SES was not associated with SOC-CA at baseline nor over time, a statistically significant gene-environment interaction was found over the 9-year study period. Specifically, participants who scored high on a polygenetic measure for cognitive ability and reported high childhood SES demonstrated a faster rate of decline in SOC-CA. These findings indicate that inter-individual differences stemming from early life influence people’s SOC-CA as they age. Overall, results suggest the importance of subgroup differences within efforts to engage individuals in preventive measures to optimize healthy brain aging.

